# Serum Pancreatic Stone Protein Reference Values in Healthy Pregnant Women: A Prospective Cohort Study

**DOI:** 10.3390/jcm12093200

**Published:** 2023-04-29

**Authors:** Ladina Vonzun, Romana Brun, Nora Gadient-Limani, Marcel André Schneider, Theresia Reding, Rolf Graf, Perparim Limani, Nicole Ochsenbein-Kölble

**Affiliations:** 1 Department of Obstetrics, University Hospital of Zurich, Frauenklinikstrasse 10, 8091 Zurich, Switzerland; 2 Faculty of Medicine, University of Zurich, Rämistrasse 71, 8091 Zurich, Switzerland; 3 Department of Obstetrics and Gynaecology, Cantonal Hospital Baden, 5404 Baden, Switzerland; 4 Department of Surgery & Transplantation, Swiss Hepatopancreatobiliary Laboratory, University Hospital Zurich, Raemistrasse 100, CH-8091 Zurich, Switzerland

**Keywords:** PSP, pancreatic stone protein, pregnancy, reference values

## Abstract

Background: In non-pregnant populations, pancreatic stone protein (PSP) has been reported to have a higher diagnostic performance for identifying severe inflammatory and infectious disease than other established biomarkers. Objective: To generate reference values for serum PSP in pregnancy and compare them to the values of the general healthy population. Design: A prospective cohort study. Setting: A single center. Population: Healthy women with singleton and multiple pregnancies. Methods: This is a prospective single-center cohort study. Between 2013 and 2021, samples of 5 mL peripheral blood were drawn from 440 healthy pregnant women. Therein, 393 cases were singletons and 47 were multiple pregnancies. Serum PSP levels were measured by specific enzyme-linked immunosorbent assay. The main outcome measures were serum PSP level (ng/mL) reference values in healthy pregnant women. Results: The mean PSP reference values in women with singleton pregnancies were 7.9 ± 2.6 ng/mL (95% CI; 2.69–13.03 ng/mL). The PSP values in women with multiple pregnancies (9.17 ± 3.06 ng/mL (95% CI; 3.05–15.28 ng/mL)) were significantly higher (*p* = 0.001). The PSP values in the first trimester (6.94 ± 2.53 ng/mL) were lower compared to the second (7.42 ± 2.21 ng/mL) and third trimesters (8.33 ± 2.68 ng/mL, *p* = 0.0001). Subgroup analyses in singletons revealed no correlations between PSP values, maternal characteristics, and pre-existing medical conditions. Conclusion: The PSP values in healthy pregnant women (4–12 ng/mL) were in the range of the reference values of the general healthy population (8–16 ng/mL). This insight blazes a trail for further clinical studies on the use of PSP as a potential novel biomarker for the early detection of pregnancy-related diseases such as chorioamnionitis.

## 1. Introduction

Pancreatic stone protein (PSP) is a C-type lectin protein that triggers polymorphonuclear cell activation and has shown proinflammatory activity in vitro [1]. Under physiological conditions, pancreatic stone protein (PSP) is predominantly produced in the pancreas and gut. Both preclinical and clinical trials demonstrated increased PSP levels in inflammatory diseases with or without infection [1,2,3]. Current evidence shows that PSP is an accurate diagnostic and prognostic marker in critically ill patients and helps in discerning the risk of developing sepsis, the severity of peritonitis, and in predicting mortality in intensive care unit patients [1,2,4,5,6]. The diagnostic performance of PSP, alone and in combination with other markers or clinical scores, was evaluated further in several studies conducted in adults, children, and neonates, in both intensive care units (ICUs) and emergency departments [7]. PSP has been reported to have a higher diagnostic performance in identifying sepsis than other established biomarkers such as procalcitonin (PCT), interleukin 6 (IL-6), and C-reactive protein (CRP) [7,8]. Despite extensive research, no novel biomarkers have been identified in order to detect sepsis in an early clinical stage and with a high degree of diagnostic accuracy. In contrast, CRP is a clinically well-characterized inflammatory marker widely measured in the diagnosis of infectious diseases. Additionally, PCT has been evaluated in the last decade as a marker of bacteremia. Even if CRP and PCT are commonly used in the context of the diagnosis of sepsis, both have shown suboptimal performance [8,9]. Combining PSP with PCT in a bio-score significantly improves the ability to diagnose neonatal early-onset sepsis [10]. So far, only one study exists regarding PSP in pregnant women, postulating PSP to be a suitable marker for the assessment of renal function in pregnancy [11].

PSP reference values of 8–16 ng/mL have been described in general non-obstetric healthy populations [9]. Increased PSP values up to 20–50 ng/mL have been observed in patients with benign disorders such as diabetes mellitus [12]. In patients with septic conditions PSP exceeds 100 ng/mL [1].

Since its first description as a potential infection biomarker in patients after trauma, PSP has repeatedly been shown to perform superiorly to CRP, and at least as well as PCT, in identifying patients with infectious diseases [1,6]. In contrast to PCT, no cut-off threshold value has yet been defined for its clinical use [13]. A systematic review obtained raw data from five observational studies and performed a meta-analysis at the individual patient level in order to explore the performance of PSP in detecting infectious diseases. The eligible studies were performed in different countries and included acutely diseased patients from emergency departments or intensive care units. The resulting cohort of 631 hospitalized adult patients encompassed an important proportion (42%) of patients without infection, which makes it the largest analysis of PSP [13]. This systematic review with meta-analysis concludes that a cut-off value of 44.18 ng/mL PSP performs better than CRP or PCT across the considered studies. The combination of PSP with CRP further enhances its accuracy [13]. 

Additionally, PSP was assessed to differentiate between burn- and infection-related inflammation [2]. Burn victims’ state of hyperinflammation triggers infectious complications. However, microbiological cultures provide results with a delay of 48–72 h following the sampling of patient material. Both of these factors postpone the initiation of targeted antimicrobial and intensive care therapy. Blood biomarkers are supposed to support the clinician’s diagnostic and therapeutic decision-making processes but often fail in that respect due to lack of sensitivity, specificity, availability or affordability. With regard to this, altered levels of pro-inflammatory markers secondary to trauma or surgery still present a major problem in that they are prone to interfere with the clinical identification of infectious events [2]. In another study, the first 14 days of serum PSP in a cohort of 90 severely burned patients (≥15% total body surface area) were analyzed to assess PSP-discriminatory accuracy in order to differentiate sterile systemic inflammation from infectious/septic clinical courses as compared to current clinically established inflammatory biomarkers (WBCs, CRP, PCT) [2]. This study evaluated the temporal course of PSP serum levels before the clinically diagnosed septic event. It concludes that PSP demonstrates high discriminatory ability in the timely identification of evolving sepsis and septic shock in patients with acute severe burns. Its steep increase allows sepsis detection up to 72 h before clinically overt deterioration, thus outperforming CRP- and PCT-based protocols for sepsis diagnosis [2]. 

To date, no reference values for pregnant women have been reported. Since pregnancy features complex immunological conditions, pregnant women are more susceptible to infections and inflammatory diseases [14,15]. Therefore, their PSP values might differ from those measured in general healthy populations. 

The aim of this study was to evaluate physiological serum levels of the novel biomarker PSP throughout different time points in singleton and multiple pregnancies and to compare these values to the reference values of the general healthy population. 

## 2. Methods

This is a prospective, single-centered cohort study assessing reference values of PSP in pregnancy.

### 2.1. Study Population

From 2013 to 2021, 440 healthy pregnant women were recruited. According to the study protocol, a minimum of seven women per week of gestation were included for determination of the reference values. 

Healthy women with a singleton (N = 393) or multiple (N = 47) pregnancy, and who were older than 18 years, were included in this study. Each woman was included once only. Women with pathological conditions such as premature ruptures of the membranes (PPROM), amniotic infection syndrome (AIS), preeclampsia (PE), and viral (hepatitis B virus, hepatitis C virus, human immunodeficiency virus or coronavirus) or confirmed bacterial infections were excluded. Information on demographics, maternal age, body mass index (BMI), week of gestation, parity, pregnancy history, and comorbidities was collected.

### 2.2. Generation of Reference Values for Pregnant Women

The generation of PSP reference values in healthy pregnant women was based on the values retrieved from the healthy singleton pregnancies of this study population. Outliers were previously excluded as per outlier testing (N = 3). 

In a second stage analysis, PSP values from multiple pregnancies were compared to reference values of the general healthy population (median: 10.8 ng/mL, interquartile range (IQR): 9.0–12.5) previously published by Schlapbach et al. [9].

### 2.3. Sample Collection and Processing

Peripheral blood (5 mL) was drawn from each participant. Following the arrival of the blood sample in the laboratory, serum PSP levels were measured by enzyme-linked immunosorbent assay (ELISA), as previously described [1,16]. All laboratory measurements were performed in triplicates. Excessive material was stored and catalogued in a central, anonymous biobank (at −80 °C temperature). 

### 2.4. Data Analysis

Statistical significance was defined as *p* < 0.05 and all tests were 2-sided. Numerical variables were summarized as mean ± standard deviation (SD) or median with interquartile range (IQR) as appropriate and were compared by Student’s t-test/ANOVA or Wilcoxon’s rank-sum test/Kruskal–Wallis test as appropriate. The normal distribution of data was evaluated with the Shapiro–Wilk test. A multivariate regression analysis was performed where necessary. The correlation between numerical variables was assessed with Pearson’s correlation coefficient. Outliers were identified using the boxplot method (above Q3 + 1.5xIQR or below Q1—1.5xIQR) with extreme outliers defined as above Q3 + 3xIQR or below Q1—3xIQR. Categorical variables are presented as number (N) and percentage (%), and were compared with Fisher’s exact test. R V4.0.2 and R-Studio V1.3.1093 (R Foundation for Statistical Computing, Vienna, Austria) were used for statistical analyses, calculations, and graphical representations.

### 2.5. Ethics Approval and Registration 

The study was conducted in accordance with the approval of the local Ethic Commission (KEK-ZH-No. 2014-0046.). Written consent was received from all women before participating in this study. Primary international registry Clinicaltrials.gov ID NCT02247297, Swiss National Clinical Trials Portal SNCTP000000290. 

## 3. Results

Baseline demographics and characteristics of the study population are shown in Table 1. 

### 3.1. Singleton Pregnancies

**Figure 1:**
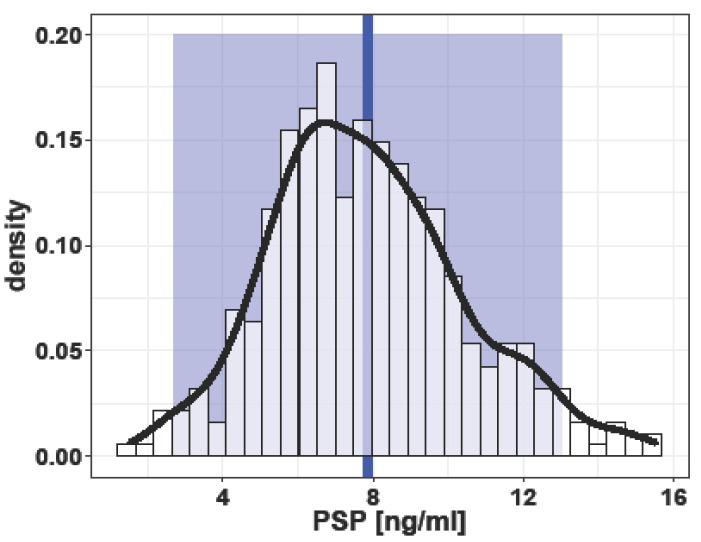
Density plot of pancreas stone protein (PSP) serum values in singleton pregnancies independent of the trimester of pregnancy. Blue line: mean overall PSP value 7.86 ng/mL. Blue square: 95% confidential interval (CI) (2.69 to 13.03 ng/mL). Black curve: density curve.

Mean PSP values in all healthy singleton pregnancies were 8.15 ± 4.23 ng/mL with a median of 7.66 (IQR: 6.10, 9.50) and were right-skewed (*p* ≤ 0.001) due to three outliers >40 ng/mL without apparent clinical reason or explanation by the analysis procedure for these high values. Of the remaining 390 patients, mean PSP values were 7.86 ± 2.59 ng/mL (95% CI; 2.69–13.03 ng/mL) (Figure 1). 

**Figure 2:**
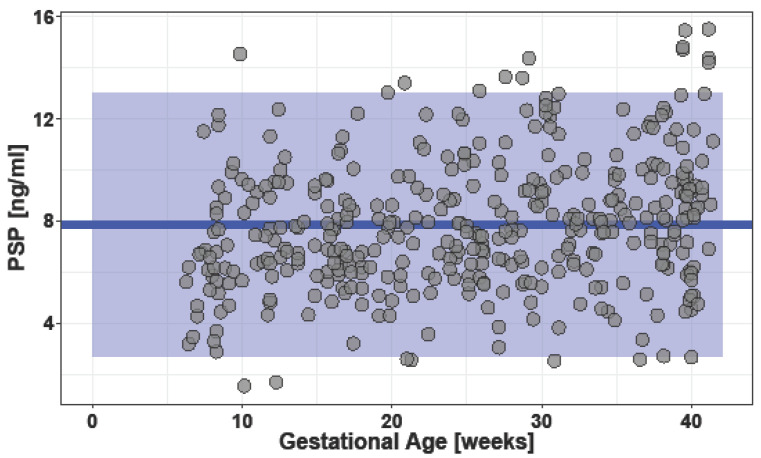
Scatter plot of pancreatic stone protein (PSP) values in singleton pregnancies according to gestational age (3 outliers not displayed). Blue line: mean overall PSP value 7.86 ng/mL. Blue square: 95% confidential interval (CI) (2.69 to 13.03 ng/mL).

Figure 2 shows the course of PSP levels in singleton pregnancies throughout pregnancy.

**Figure 3:**
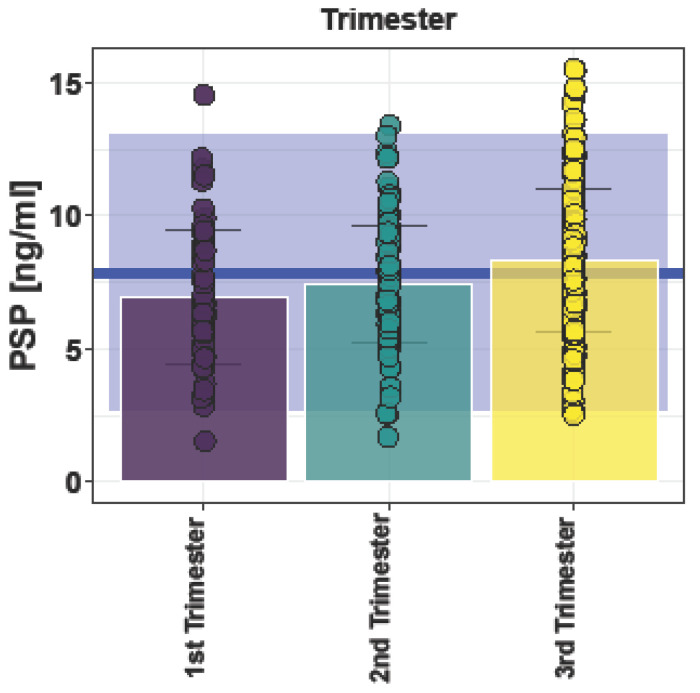
Bar plot of pancreatic stone protein (PSP) values in singleton pregnancies grouped according to trimester (Mean ± standard deviation (SD)). 1. Trimester 6.96 ± 2.5 ng/mL, 2. Trimester 7.43 ± 2.21 ng/mL, 3. Trimester 8.34 ± 2.69 ng/mL. Blue line: mean overall PSP value 7.86 ng/mL. Blue square: 95% confidential interval (CI) (2.69 to 13.03 ng/mL).

PSP values in the first trimester (6.94 ± 2.53 ng/mL) were lower compared to the second (7.42 ± 2.21 ng/mL) and third trimesters (8.33 ± 2.68 ng/mL, *p* = 0.0001) (Figure 3). 

A comparison of gestational age to PSP level showed a correlation (R = 0.22, *p* ≤ 0.0010). 

No correlation between PSP values and BMI (R = −0.06, *p* = 0.22) or maternal age (R = 0.08, *p* = 0.11) was observed. Furthermore, no statistical difference in PSP values and women’s parity (*p* = 0.59) and ethnicity (*p* = 0.50), nor in women with and without comorbidities, was noted (Table 2). 

### 3.2. Multiple Pregnancies

**Figure 4:**
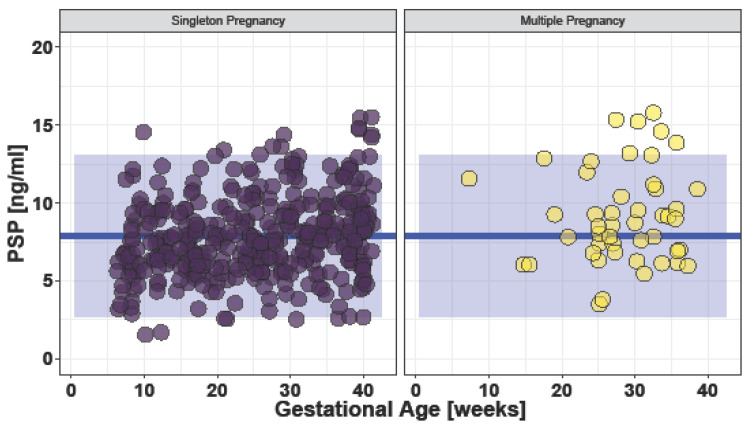
Bar plot of PSP values grouped in singleton vs. multiple pregnancies (3 outliers not displayed). Blue line: mean overall PSP value in healthy pregnant women 7.86 ng/mL. Blue square: 95% CI (2.69 to 13.03 ng/mL).

Mean PSP values in the 47 healthy women with multiple pregnancies were 9.17 ± 3.06 ng/mL (95% CI; 3.05–15.28 ng/mL). No difference in PSP values in the different trimesters (*p* = 0.69) was observed and PSP levels showed no correlation to gestational age (R = 0.075, *p* = 0.62). Subgroup analysis revealed that maternal age is correlated with PSP values in women with multiple pregnancies (R = 0.31, *p* = 0.04). No relevant influence of further baseline characteristics and comorbidities on PSP levels were found in multiple pregnancies. Furthermore, no difference in the chorionicity of the multiple pregnancies and PSP values was observed (*p* = 0.52). However, PSP serum values were significantly increased in women with multiple pregnancies compared to singleton pregnancies (*p* = 0.001) (Figure 4).

A comparison of PSP serum values in healthy pregnant women with singleton (median: 7.66 ng/mL, IQR: 6.10, 9.50) and multiple (median: 8.73 ng/mL, IQR: 6.88, 11.1) pregnancies with reference values of the general healthy population (median: 10.8 ng/mL, IQR: 9.0–12.5) revealed no relevant differences (*p* = 0.21 and *p* = 0.52, respectively).

## 4. Discussion

### 4.1. Main Findings

This study reports the physiological course of PSP serum values in healthy pregnant women. The reference values obtained in women with singleton 8 ± 3 ng/mL and multiple 9 ± 3 ng/mL pregnancies are comparable to the values in the healthy general population of 8–16 ng/mL [9]. Therefore, this study blazes a trail for the potential use of PSP as a novel biomarker in pregnant women in corresponding clinical situations, such as inflammatory or infectious diseases.

### 4.2. Interpretation

PSP was introduced as a potential infection biomarker in patients after trauma (Ref: Keel, 2009). Since then, several studies have repeatedly shown that PSP performs superiorly to CRP and at least as well as PCT in identifying patients with infectious diseases [1,6]. A systematic review obtained raw data from five observational studies and carried out a meta-analysis at the individual patient level to explore the performance of PSP in detecting infectious diseases. The conclusion drawn across the considered studies is that, at a cut-off value of 44.18 ng/mL, PSP performs better than CRP or the combination of PSP with CRP further enhances its accuracy [13]. The diagnostic accuracy for the diagnosis of sepsis of PSP, CRP, and PCT were reported to be similar [8]. They report that serial measurements of biomarker revealed that blood PSP levels increased incrementally 3 days before the clinical diagnosis of nosocomial sepsis in critically ill patients, potentially allowing the early detection of sepsis before the appearance of signs and symptoms [8]. Some aspects of the clinical use of PSP deserve detailed considerations. Knowledge about norm values of PSP is of importance since the performance of various biomarkers (e.g., leucocytes) differs in the case of pregnancy compared to in the general population due to modified immune response in pregnant women [17]. The immune system of pregnant women is modulated by complex mechanisms to allow the genetically and immunologically foreign fetus to thrive and prosper [17]. Consequently, the immunologic response in pregnancy is altered leading to decreased stress response [17] which might cause altered PSP values throughout pregnancy. On the other hand, immunological modifications render pregnant women more susceptible to infections [14,15,17].

A further aspect is the increased glomerular filtration rate in pregnancy [18]. PSP, being a low molecular weight protein of 14–19 kilodaltons (kDa), crosses the glomerular basement membrane and undergoes reabsorption in the proximal renal tubules [19]. Whereas glomerular filtration rate is known to increase throughout pregnancy, reabsorption in the proximal renal tubes remains constant [17]. This renal loss of PSP could result in the rather low values in comparison with the known values of the general population. 

Another consideration is that our study shows a significant rise in PSP values along with increasing gestational age. This observation might be important in terms of the interpretation of PSP values as a novel biomarker in pregnancy. Schlapbach et al. reported relevant age-dependent variations of PSP values and emphasized the practical need for different thresholds for PSP [9]. In this context, it might be possible that PSP thresholds for detecting pregnancy-related diseases may even need to be adapted during the course of pregnancy, even if the differences in references between the trimesters found in this study were clinically irrelevant

Moreover, PSP values observed in healthy pregnant women did not show any correlation with pre-existing non-infectious medical conditions such as cardiac, autoimmune, renal, and diabetic conditions. Earlier studies revealed that urinary PSP values increase markedly in patients with various renal diseases, particularly also in diabetic nephropathies [19]. At the same time, PSP serum levels were described to rise in patients with diabetes mellitus [12,16]. However, these observations were independent from the glomerular filtration rate and, rather, led back to an augmented PSP secretion by the kidney itself as well as an inflammatory response of the infiltrated beta cells of the pancreas [12,16]. We could not undermine these observations. Eventually, in our cohort, the close follow-up and the rigorous control of pre-existing medical conditions as well as gestational diabetes during pregnancy, may explain our findings. The sole correlation observed was the correlation with PSP levels and maternal age in multiple pregnancies. To date, we do not have a plausible explanation for this observation. However, there is no clinical relevance of the absolute value differences.

### 4.3. Strengths and Limitations

This study presents PSP norm references in the first prospective cohort of healthy pregnant women. PSP values were measured under clearly defined clinical and laboratory criteria, conditions under which data are reproducible. Based on this large dataset, we report that the novel biomarker PSP measured in pregnant women is comparable to values measured in the general healthy population. This study, however, does not answer the question of whether PSP really holds its promise as a novel clinical biomarker in obstetric pathological conditions and is clearly not designed to provide thresholds or cut-offs for pathologies. Therefore, we are currently designing further clinical trials to evaluate PSP values in pregnant women diagnosed with pathological diseases. 

## 5. Conclusions

PSP serum values in healthy pregnant women (4–12 ng/mL) correspond to the reference values of the general population (8–16 ng/mL). This insight blazes a trail for further clinical studies on the use of PSP as a potentially novel biomarker for early detection of pregnancy-related conditions such as chorioamnionitis.

## Figures and Tables

**Table 1:** Baseline characteristics of singleton and multiple pregnancies.

	Singleton Pregnancy (N = 390)	Multiple Pregnancy (N = 47)	*p*-Value	Total (N = 440)
PSP				
Mean ± SD [ng/m]	7.86 ± 2.59	9.17 ± 3.06	0.001	8.26 ± 4.13
Maternal Age				
Mean ± SD [years]	32.9 ± 5.29	35.1 ± 4.17	0.001	33.1 ± 5.22
Parity				
Mean ± SD	2.12 ± 1.34	2.32 ± 1.32	0.33	2.14 ± 1.34
BMI				
Mean ± SD [kg/m^2^]	23.8 ± 5.19	24.2 ± 4.87	0.59	23.8 ± 5.15
Ethnicity [N (%)]			0.42	
Afro-Caribbean	17 (4.4%)	2 (4.3%)		19 (4.3%)
Asian	23 (5.9%)	1 (2.1%)		24 (5.5%)
Caucasian	283 (72.1%)	40 (85.1%)		323 (73.4%)
Mediterranean	38 (9.7%)	4 (8.5%)		42 (9.5%)
Mixed	7 (1.8%)	0 (0.0%)		7 (1.6%)
Oriental	24 (6.2%)	0 (0.0%)		24 (5.5%)

Abbreviations: pancreatic stone protein (PSP), standard deviation (SD), body mass index (BMI).

**Table 2:** Comparison between pancreatic stone protein (PSP) in 390 healthy singleton pregnancies with and without comorbidities.

Comorbidities	Singleton Pregancy [N (%)]	PSP Values Mean ± SD [ng/m]	*p*-Value
Thyroid disorders			
Hypothyroidism	18 (4.6%)	7.10 ± 2.51	0.43
Hyperthyroidism	3 (0.8%)	10.45 ± 3.41	0.20
no	369 (94.6%)	7.87 ± 2.57	
Gestational diabetes			
Diet	64 (16.3%)	6.76 ± 1.91	0.99
Insulin therapy	8 (2.1%)	7.84 ± 2.78	0.44
no	318 (81.5%)	7.89 ± 2.56	
Cardiac disease			
yes	13 (3.3%)	8.38 ± 1.81	0.45
no	377 (96.7%)	7.84 ± 2.61	
Rheumatologic disease			
yes	8 (2.1%)	7.31 ± 2.06	0.55
no	382	7.87 ± 2.60	
Hematologic disease			
yes	13 (3.3%)	8.10 ± 2.40	0.73
no	377 (96.7%)	7.85 ± 2.59	
Nicotine Abuse			
yes	13 (3.3%)	8.10 ± 2.40	0.73
no	377 (96.7%)	7.85 ± 2.59	

## Data Availability

Data are available in the main text.

## Abbreviations

AIS	amniotic inflammatory syndrome
BMI	Body mass index
CRP	C-reactive protein
ELISA	Enzyme Linked Immunosorbent Assay
PE	preeclampsia
IL-6	interleukin 6
IQR	Interquartile range
PCT	procalcitonin
PSP	Pancreatic stone protein
SD	Standard deviation
SNCTP	Swiss National Clinical Trials Portal
